# Non-pharmacological mental health interventions for older adults in Mexico: a systematic review

**DOI:** 10.3389/fragi.2026.1730672

**Published:** 2026-02-23

**Authors:** María Fernanda Zapata-De la Rosa, Harvey Apolonio-Cortés, Rodrigo Ramirez-Rodriguez, Rafael Fernández-Demeneghi, Yuliana Yessy Gomez Rutti, Fabiola Ortiz-Cruz, Angel Puig-Lagunes

**Affiliations:** 1 Faculty of Medicine, Universidad Veracruzana, Minatitlan Campus, Veracruz, Mexico; 2 Researcher of National Polytechnic Institute, Mexico, Mexico; 3 Institute for Research on Eating Behavior and Nutrition, University of Guadalajara, Ciudad Guzmán, Mexico; 4 Private University In The North, Lima, Peru; 5 Faculty of Dentistry, Veracruz University, Minatitlan, Mexico

**Keywords:** mental health, aged, complementary therapies, geriatric psychiatry, health promotion

## Abstract

**Introduction:**

The accelerated aging of the Mexican population presents an urgent public health challenge, particularly regarding geriatric mental health. While non-pharmacological interventions (NPIs) offer a promising therapeutic avenue, national evidence remains fragmented. This systematic review critically evaluates the efficacy of NPIs implemented in Mexico to improve mental health outcomes among adults aged 60 and older.

**Method:**

Adhering to PRISMA 2020 guidelines and registered in PROSPERO (CRD420251033051), we conducted a comprehensive search across PubMed, Scopus, and the Virtual Health Library (2010–2025). We included randomized controlled trials and quasi-experimental studies, assessing risk of bias via Joanna Briggs Institute tools.

**Results:**

Seven studies (N = 267; mean age 71.9 ± 7.3) met the eligibility criteria. Synthesis of findings revealed distinct efficacy patterns: physical exercise interventions yielded the most robust outcomes, demonstrating large effect sizes for reducing depressive symptoms (ηp^2^ = 0.35) and enhancing resilience (ηp^2^ = 0.46). In contrast, cognitive and reminiscence-based therapies proved highly effective for improving self-esteem (d = −0.89) but showed inconsistent results for mood regulation.

**Discussion and Conclusion:**

Current evidence confirms that NPIs—specifically structured physical activity—are potent and scalable tools for promoting geriatric mental health in Mexico. However, the existing literature is limited by heterogeneity and a lack of geographic coverage. To bridge the gap between research and practice, it is imperative that policymakers transition from isolated pilot interventions to the integration of standardized, evidence-based NPIs protocols within the national geriatric care system.

**Systematic Review Registration:**

https://www.crd.york.ac.uk/PROSPERO/view/CRD420251033051, identifier CRD420251033051.

## Introduction

Aging is a physiological, individual, and heterogeneous process influenced by social, health-related, economic, and cultural factors (Congreso General de los Estados Unidos Mexicanos, 2024). Mexican legislation and international organizations define older adults (OAs) as individuals aged 60 years or older (Estebsari et al., 2020). Projections indicate that the global geriatric population will double by 2050 (United NationsDepartment of Economic and Social AffairsPopulation Division, 2019). In Mexico, the OAs population is projected to reach 20.6 million by 2030, representing 15% of the total population (Instituto Nacional de las Personas Adultas Mayores, 2024). However, this demographic shift contrasts sharply with the limited availability of specialized care, as in 2025, there was only one certified geriatrician per 13,000 OAs (Consejo Mexicano de Geriatría, 2025). The marked heterogeneity of aging increases vulnerability to developing affective disorders, with reported prevalence rates of anxiety and depression of 28% and 39%, respectively (Estebsari et al., 2020; De la Vega Martínez et al., 2023).

These conditions are closely associated with functional decline, frailty, worsening of comorbidities, and a consequent reduction in quality of life (Coveñas et al., 2024). Furthermore, substantial gaps in healthcare coverage, infrastructure, medication supply, and mental health service availability persist for OAs in Mexico (Juárez et al., 2020). As a result, up to 96.8% of this population does not receive treatment. This gap is closely linked to low health service utilization, largely driven by structural barriers such as geographic distance and limited access to transportation (Guzmán-Olea et al., 2018).

In this context, non-pharmacological interventions (NPIs) emerge as a particularly relevant strategy, given their demonstrated benefits on emotional wellbeing, functional capacity, and quality of life, as well as their favorable cost-effectiveness, safety profile, sustainability, and replicability (Gramaglia et al., 2021; Apóstolo et al., 2016). Evidence from systematic reviews and meta-analyses indicates that a broad range of NPIs, including physical activity, horticulture, cognitive behavioral therapy, positive reminiscence, mindfulness-based interventions, and others, may be more effective than pharmacological treatment in reducing depressive symptoms (Gramaglia et al., 2021; Apóstolo et al., 2016; Chen et al., 2021; Frost et al., 2019). These interventions have been associated with reductions of 20%–30% in depressive symptoms and improvements of 10%–20% in life satisfaction, with sustained benefits lasting up to six to 12 months, while avoiding adverse effects, pharmacological dependence, and constraints related to medication availability or economic factors.

Due to the steady increase in the geriatric population and disparities in the availability of and access to mental health services, implementing evidence-based policies, particularly NIPs, is a fundamental public health strategy to mitigate these deficiencies among OAs in Mexico. This review aims to identify NIPs implemented in Mexico to promote, maintain, and improve OAs mental health. Additionally, it will examine the main challenges and opportunities for replicating these policies.

## Methods

### Information sources and search strategy

The protocol was registered in PROSPERO (CRD420251033051) and conducted in accordance with the PRISMA 2020 guidelines (Page et al., 2021). Using the PICo structure (Munn et al., 2018) and MeSH terms with Boolean operators, we searched PubMed, Scopus, and the Virtual Health Library from 1 January 2010, to 3 February 2025. Population: “Mexican older adults”; Intervention: “No-pharmacological intervention”; Comparison: “No-intervention group”; Outcome: “Psychological symptoms.” The following MeSH terms were employed in the search strategy: Elderly OR “Older adults” OR “Mexican older adult” AND “Mental health assistance” OR “Mental health services” OR “Healthy aging” OR “Intervention program” OR Program OR Intervention OR Strategy OR Project AND “Mental health” OR “Health, Mental”.

### Eligibility criteria

Studies published in Spanish or English and conducted in Mexico were included. The populations consisted of OAs aged 60 years or older. Randomized clinical trials and quasi-experimental studies were considered. These studies evaluated NPIS aimed at promoting, maintaining, or improving mental health in conditions such as depression, anxiety, stress, and other mood states.

Systematic reviews with or without meta-analyses, narrative reviews, bibliometric studies, gray literature, opinions, and letters to the editor were excluded. Studies focusing exclusively on people with neurocognitive disorders (e.g., dementia, Alzheimer’s disease, or cognitive impairment) were excluded. Exceptions were made for studies with a mixed population if OAs data on healthy ageing could be extracted independently.

### Selection process

The records were imported into the Rayyan web application for systematic reviews (Ouzzani et al., 2016). Two reviewers (HA-C and MFZ-R) independently and in a blinded manner performed title and abstract screening. Any discrepancies were resolved through consensus with a third reviewer, who reviewed the full texts and applied the previously established inclusion and exclusion criteria (AAP-L).

### Data collection and data items

Following study selection, a Microsoft Excel matrix was designed for data extraction and included the following categories: 1) author, 2) objective, 3) methods, 4) intervention results, and 5) challenges and opportunities.

### Synthesis methods

A narrative synthesis of the findings was conducted. Studies were grouped by intervention type and assessed outcomes.

### Methodological quality assessment

We evaluated the methodological quality of the included studies using the Joanna Briggs Institute (JBI) checklists for randomized controlled trials (Barker et al., 2023) and quasi-experimental studies (Barker et al., 2024). These tools are designed to assess the methodological rigor of various quantitative study designs. To ensure a comprehensive appraisal of all available evidence, no cutoff score was applied for study exclusion.

### Ethics statement

This study is a systematic review of previously published studies and uses only aggregated, non-identifiable data. Therefore, ethical approval and informed consent were not required.

## Results

### Study selection

**FIGURE 1:**
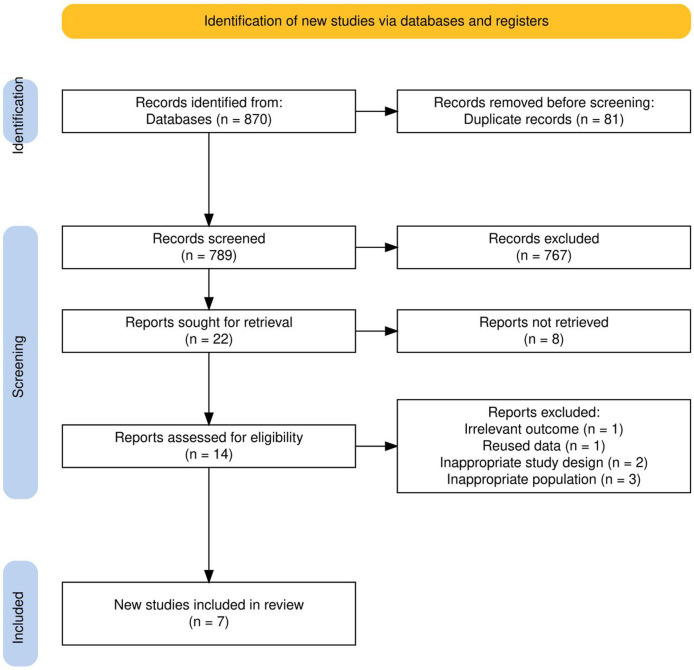
Flow chart of the study selection process.

The search strategy yielded 870 records. The Rayyan application detected 154 potential duplicates; 81 of these were confirmed and eliminated. This left 789 unique records for the screening phase. After reviewing the titles and abstracts, 22 studies were selected for full-text evaluation. Eight studies were included by consensus, while 14 records were subject to discrepancies. A third reviewer resolved the discrepancies, resulting in the inclusion of six additional studies. Seven studies ultimately met the eligibility criteria (see Appendix for excluded studies) (Figure 1).

### Study characteristics

Four studies had a quasi-experimental design, while two others used a randomized controlled trial design. One study was described as a pragmatic clinical trial. The studies were conducted in Durango, Sonora, Baja California, and Mexico City. Seventy-one percent (*n* = 5) of the studies were conducted in non-institutional settings in Mexico. The remaining studies took place in private (14.2%, *n* = 1) (25) and government (14.2%, *n* = 1) institutions. Table 1 summarizes the main characteristics of these studies.

**TABLE 1:** Characteristics of the studies on non-pharmacological interventions included.

Author, year	Objective	Method	Results
Villarreal-Ángeles et al. (2016)	To determine the effect of a physical conditioning program based on the pilates method on mood variables in OAs in the state of durango, Mexico	Design and sample: Experimental study with pre- and post-intervention measurements in 20 non-institutionalized OAs from durango without functional limitations, divided into CG: *n* = 10 and EG: *n* = 10 Average age: 60–80 years. GE: 65.0 ± 3.3, CG: 69.3 ± 7.4 Sex: 5 males (EG: 2, CG: 3), 15 females (EG: 8, CG: 7) Intervention: 12-week pilates-based exercise program, with three 50-min sessions per week, structured in three phases: Physical and respiratory preparation (5 min), exercise routine with breaks (40 min), and relaxation with final feedback (5 min) Assessment instrument: POMS	Significant reduction in the dimensions of tension (pre: 2.5 ± 2.3 vs. post:1.4 ± 1.5, *p ≤* 0.001), anger (pre: 1.8 ± 2.9 vs. post: 0.8 ± 1.5, *p =* 0.030), fatigue (pre: 3.3 ± 3.3 vs. post: 1.2 ± 1.9, *p ≤* 0.01), and total POMS score (pre: 10.5 ± 9.1 vs. post 5.9 ± 5.3 1, *p ≤* 0.001) No significant interaction was found in the dimensions of depression (pre: 0.7 ± 1.9 vs. post: 0.3 ± 07, *p =* 0.065) or vigor (pre: 8.4 ± 2.6 vs. post: 9.8 ± 1.3, *p =* 0.125)
Borbón-Castro et al. (2020)	Investigate the effects of a 12-week multidimensional exercise program on the health behavior and biopsychological factors of OAs living in northeastern Mexico	Design and sample: Quasi-experimental study with pre- and post-intervention measurements in 45 non-institutionalized OAs without functional limitations from sonora, divided into: CG: *n* = 23 and EG: *n* = 22 Average age: GE: 67.7 ± 6.6 years and cg: 66.6 ± 4.6 Sex: EG: 18 female and 5 male; CG: 17 female and 5 male. Total sample: 77.7% (*n* = 35) female and 22.2% (*n* = 10) male Intervention: Multidimensional exercise program, lasting 12 weeks, 5 days a week for 60 min, structured in three phases: Warm-up (10 min), exercise (40 min), and cool-down (10 min) Assessment instrument: GDS-15 and RSES	Reduction in depressive symptoms between the pre- and post-intervention groups (*p ≤* 0.001). Depression in the EG (pre: 1.91 ± 2.60 vs. post: 0.60 ± 1.03; *p =* 0.01) In terms of self-esteem, significant differences were observed between the groups in the post-test (*p ≤* 0.01); the EG showed a significant increase (pre: 23.42 ± 1.50 vs. post: 24.91 ± 1.83*; p ≤* 0.01*, d =* −0.89)
Cantón-Martínez et al. (2024)	Evaluate the impact of a virtually supervised exercise program on the physical fitness and mental health of older Mexican adults during pandemic lockdown	Design and sample: Pragmatic clinical trial in 44 non-institutionalized OAs from tijuana divided into 4 groups: Healthy CG healthy: *n* = 15, CG with illness or comorbidities: *n* = 9, EG healthy: *n* = 11, EG illness: *n* = 9 Age and sex: No analyses or distributions were presented Intervention: Supervised virtual exercise program on the google Meet platform, for 12 weeks, 3 times a week, in 60-min sessions, structured in three phases: Warm-up and stretching (10 min), aerobic and progressive strength exercises (40 min), and cool-down with final stretching (10 min) Assessment instrument: HDRS-17, GDS-15 and CD-RISC	Reduction in depressive symptoms on both scales: GDS-15 (pre: 5.06 ± 2.60 vs. post: 2.00 ± 1.33; *p ≤* 0.001*; ηp* ^*2*^ *=* 0.35) and HDRS-17 (pre: 8.11 ± 4.64 vs. post: 4.38 ± 2.23; *p ≤* 0.001*; ηp* ^*2*^ *=* 0.35) and resilience scores (post: 27.2 ± 4.6 vs. post: 34.6 ± 2.01*: p ≤* 0.001*; ηp* ^*2*^ *=* 0.46)
Gómez-Miranda et al. (2019)	To determine the effect of a physical exercise program using exergames (xbox physical exercise games) on functional and cognitive capacity, depressive state, and risk of falls in older Mexican adults	Design and sample: Quasi-experimental study with pre- and post-intervention measurements in 14 non-institutionalized OAs without functional limitations in tijuana, divided into a CG (*n* = 7) and an EG (*n* = 7) Type of residence: Community Average age: >60 years. EG = 65.4 ± 6.4, CG = 65.1 ± 11.4 Sex: No analyses or distributions were presented Intervention: Physical exercise program with exergames, for 15 weeks, 3 times a week with 120-min sessions, structured in three phases: Warm-up and stretching (15 min), exergames (45 min), and cool-down (15 min). The control group performed a weekly session of conventional low-intensity physical exercise (40 min) Assessment instrument: GDS-15	No significant interactions were found between groups and measurements in the GDS (*p =* 0*.*866*, η* ^*2*^ *=* 0*.*2%). Regardless of the EG group, initial measurements were higher than final measurements in the GDS (3.93 ± 0.72 vs. 3.00 ± 0.67, p = 0.045 η^2^ = 29.5%)
Ortiz-Ortiz et al. (2019)	To determine the effects of a physical activity program on depressive symptoms and functional capacity in institutionalized OAs in tijuana, Mexico	Design and sample: Experimental, longitudinal study with pre- and post-intervention measurements in 50 OAs institutionalized in retirement homes in tijuana, divided into CG: *n* = 25 and EG: *n* = 25 Average age: EG: 73.1 ± 6.8 years and CG: 75.9 ± 5.7 years. Male EG: 73.1 ± 6.8 years, CG: 75.9 ± 5.7 years. Female EG: 75.2 ± 5.8 years, CG: 72.7 ± 6.2 years Sex: Males (*n* = 20) distributed into 10 CG and 10 EG; and females (n = 30) divided into 15 CG and 15 EG Intervention: Chair-based exercise program, for 12 weeks, 5 times per week, in sessions lasting 40–50 min, structured in three phases: Warm-up (10 min), physical exercise (25 min), and relaxation (10 min) Assessment instrument: GDS-15	A significant triple interaction (*p* ≤ 0.001, *η2* = 20.3%) was found between men and women in the CG and EG groups and pre- and post-measurements of depressive state. In male in the EG there was a reduction in depressive state (pre: 8.2 ± 6.1 vs. post: 2.9 ± 3.5), as was the case in females in the EG (pre: 8.5 ± 2.8 vs. post: 1.6 ± 2.2)
Sánchez-Nieto et al. (2019)	To determine the effect of a mental stimulation program based on learning to use computers and the internet on cognitive functions and wellbeing in older Mexican adults living in the community	Design and sample: Quasi-experimental pilot study with pre- and post-intervention measurements in 27 non-institutionalized OAs without functional limitations in Mexico City, divided into CG: n = 11 and EG: n = 16 Average age: EG: 64.3 ± 3,4 years and CG: 64.3 ± 3.2 years Sex: EG: 2 males and 14 females; CG: 3 males and 8 females Intervention: Mental stimulation program, consisting of 10 weeks of 20 sessions divided into twice-weekly sessions, for a total duration of 40 h. The sessions were based on a computer course, adapted to the personal preferences of the participants using self-directed learning and positive reinforcement Assessment instrument: PE and NE; LSS	No significant differences were observed between the EG and the CG in the scores of the negative emotions test (EG: 16.7 ± 5.6 vs. CG: 17.5 ± 6.9, *p =* 0.72*, ηp* ^*2*^ *=* 0.004); the positive emotions test (EG: 41.4 ± 6.5 vs. CG: 37.1 ± 5.5, *p =* 0.61*, ηp* ^*2*^ *=* 0.01); and the life satisfaction test (EG: 21.4 ± 1.7 vs. CG: 19.7 ± 2.5, *p =* 0.06*, ηp* ^*2*^ *=* 0.12)
Villasán-Rueda et al. (2023)	Test the effectiveness of the “positive reminiscence program” (REMPOS) on cognitive and affective variables in OAs in northern Mexico with different types of aging	Design and sample: Experimental study with pre- and post-intervention measurements in 67 OAs institutionalized in residential care centers or day centers in tijuana, divided into CG: n = 33 and EG: n = 34, distributed into healthy (CG: n = 13 and EG: n = 1), mild cognitive impairment (CG: n = 10, EG: n = 12), or Alzheimer’s disease (CG: n = 10, EG: n = 11) Average age: 76.2 ± 9,7 years. Males 77.3 ± 8.65 years and females 75.8 ± 10.1 years Sex: 51 females and 16 males in the total sample Intervention: REMPOS, over a period of 2 months, divided into 12 sessions, twice a week, with each session lasting 1 hour Assessment instrument: GDS-30 and LSI-A	A significant difference was observed after the intervention in the healthy aging, EG (pre: 7.3 ± 3.5 vs. post: 5.3 ± 3.1; *t*_*9*_ *=* 3.21*; p ≤* 0.01*; Hedges’ g =* 0.93), while the CG showed no difference (*p =* 0.74) The healthy aging EG did not show a significant difference in the LSI-A due to the intervention (pre: 26.5 ± 5.3 vs. post: 28.8 ± 6.1, *p =* 0.12)

Older Adults (OAs); Control group (CG); Experimental group (EG): Profile of Mood States (POMS); Geriatric Depression Scale (GDS-15 o GDS-30); Rosenberg Self-Esteem Scale (RSES); Hamilton Depression Scale (HDRS-17); Connor Davidson Resilience Scale (CD-RISC); positive and negative emotion (PE, and NE) Positive reminiscence program (REMPOS); Life Satisfaction Scale (LSS); Life Satisfaction Index-A (LSI-A); Senior fitness test (SFT). The results are presented uniquely by the experimental group.

### Participants

The seven studies included a total of 267 OAs, with sample sizes ranging from 14 to 67. Of those, 57.3% (*n* = 153) were women, and 20.9% (*n* = 56) were men. Two studies did not report the sex distribution of their participants, accounting for 21.7% (*n* = 58) of the total sample. Ages ranged from 60 to 91 years, with an overall mean age of 71.9 ± 7.3 years. Most studies included OAs around 65 ± 6 years old, while others considered OAs closer to 74 ± 6 years old. One study did not report the mean age.

### Types of NPIs in OAs in Mexico

The identified NPIs include in-person physical exercise programs, virtual-adapted programs, positive reminiscence therapy, and cognitive stimulation using technology.

In-person interventions featured Pilates-based exercises, a multidimensional program, physical exercise, and exergames. These activities focused on improving flexibility, speed, muscle strength, coordination, agility, postural stability, and proprioception through exercises such as group dynamics, aerobic circuits, elastic bands, and video games. The virtual program offered home-based aerobic exercises, supervised via Google Meet. The mental stimulation program focused on developing a computer course based on positive feedback and self-imposed goals, incorporating personal interests and positive reinforcement. Finally, the Positive Reminiscence intervention consisted of sessions focused on remembering and discussing significant milestones in the OAs lives.

Intervention durations ranged from 2 months to 15 weeks, with sessions occurring 2 to 5 times per week and lasting 40–120 min.

## Methodological quality in studies

Two studies were randomized controlled trials, and five were quasi-experimental. The mean scores were 5.5 (maximum 13) and 6.8 (maximum 9), respectively (See Tables 2, 3).

**TABLE 2:** Quality assessment of included studies using the Joanna Briggs Institute critical appraisal tool for randomized controlled trials.

Studies	Q1	Q2	Q3	Q4	Q5	Q6	Q7	Q8	Q9	Q10	Q11	Q12	Q13	Score
Villarreal-Ángeles et al. (2016)	Y	N	N	N	N	Y	N	Y	U	Y	Y	Y	Y	7/13
Villasán-Rueda et al. (2023)	Y	N	N	N	N	Y	N	Y	U	Y	N	N	N	4/13

Y: yes, N: no, U: unclear, NA: not applicable.

**TABLE 3:** Quality assessment of included studies using the Joanna Briggs Institute critical appraisal tool for quasi-experimental studies.

Studies	Q1	Q2	Q3	Q4	Q5	Q6	Q7	Q8	Q9	Score
Borbón-Castro et al. (2020)	Y	Y	N	Y	Y	Y	U	Y	Y	7/9
Cantón-Martínez et al. (2024)	Y	Y	N	Y	Y	Y	Y	Y	Y	8/9
Gómez-Miranda et al. (2019)	Y	Y	Y	N	Y	Y	U	Y	Y	7/9
Ortiz-Ortiz et al. (2019)	Y	Y	U	Y	Y	Y	U	U	Y	6/9
Sánchez-Nieto et al. (2019)	Y	Y	Y	U	Y	Y	U	N	Y	6/9

Y: yes, N: no, U: unclear, NA: not applicable.

## Results of NPIs in Mexico

### Depressive symptoms

Most NPIs demonstrated significant reductions in depressive symptom scores within the experimental groups at post-intervention assessments compared with pre-intervention assessments (Borbón-Castro et al., 2020; Cantón-Martínez et al., 2024; Ortiz-Ortiz et al., 2019; Villasán-Rueda et al., 2023). Specifically, some studies found significant improvements from baseline to post-intervention on the Geriatric Depression Scale (*p* ≤ 0.001; *ηp* ^*2*^ = 0.35) and Hamilton Depression Scale (*p ≤* 0.001*; ηp* ^*2*^ *=* 0.46) (Cantón-Martínez et al., 2024). In addition, other studies reported significant differences between the experimental and control groups following the intervention (Borbón-Castro et al., 2020; Villasán-Rueda et al., 2023), as well as reductions in symptoms observed in both men and women.

When considering delivery methods, personalized, face-to-face interventions showed stronger effects (Villarreal-Ángeles et al., 2016; Borbón-Castro et al., 2020; Gómez-Miranda et al., 2019; Ortiz-Ortiz et al., 2019). By contrast, virtual programs yielded smaller effect sizes compared to face-to-face interventions (Cantón-Martínez et al., 2024; Sánchez-Nieto et al., 2019). Furthermore, some studies found no significant changes (Villarreal-Ángeles et al., 2016; Gómez-Miranda et al., 2019; Ortiz-Ortiz et al., 2019), and one noted increased symptoms among women (Ortiz-Ortiz et al., 2019).

### Self-esteem

A multidimensional exercise program, including aerobic, strength, and flexibility activities, showed a significant increase in self-esteem in the experimental group compared with baseline (*p* = 0.005) (Borbón-Castro et al., 2020).

### Resilience

A virtually supervised exercise program, consisting of live online aerobic and resistance sessions, revealed a significant improvement in resilience scores in the experimental group after the intervention (*p* ≤ 0.001; *ηp* ^2^ = 0.46) (Cantón-Martínez et al., 2024).

#### Mood

The Pilates-based exercise program, focused on mat exercises and flexibility training, showed a reduction in tension (*p* ≤ 0.001), anger (*p* = 0.030), fatigue (*p* = 0.002), and the total POMS score (*p* ≤ 0.001); however, it did not produce significant changes in vigor (*p* = 0.125) (Villarreal-Ángeles et al., 2016).

### Psychological wellbeing and life satisfaction

A mental stimulation program (Sánchez-Nieto et al., 2019), which involved cognitive games and memory tasks, and a positive reminiscence program (Villasán-Rueda et al., 2023), which used guided recall of pleasant life events, showed no significant differences in positive affect (*p* = 0.61), negative affect (*p* = 0.72), or satisfaction (*p* = 0.06) between the experimental and control groups after the intervention.

## Discussion

To our knowledge, this is the first systematic review conducted in the Mexican population to evaluate the comprehensive impact of NPIs on OAs mental health. Overall, our findings indicate that structured physical exercise programs are consistently associated with reductions in depressive symptoms and improvements in resilience (Borbón-Castro et al., 2020; Cantón-Martínez et al., 2024; Ortiz-Ortiz et al., 2019). These effects appear to be modulated by baseline depressive severity and participants’ cognitive and functional status, with greater benefits observed in group-based interventions tailored to OAs’ preferences and delivered under multidisciplinary supervision. Intervention adherence, duration, and individualization emerged as critical determinants of effectiveness, underscoring the importance of adaptive program design in this population (Niclasen et al., 2018; Gramaglia et al., 2021; Athanasiou et al., 2023; Santoyo-Sánchez and Reyes-Morales, 2024).

Beyond descriptive associations, emerging mechanistic evidence provides a coherent biological and conceptual framework to explain the mental health benefits of exercise-based NPIs. Recent integrative studies indicate that physical activity enhances neural connectivity and neurogenesis, modulates stress-regulatory systems by reducing cortisol and other stress-related hormones, and promotes the release of neurotransmitters involved in mood regulation and emotional resilience. In parallel, exercise promotes muscle regeneration and attenuates systemic inflammation and oxidative stress. Collectively, these mechanisms help explain why exercise-based NPIs may be more effective in improving mood, attention, executive function, memory, quality of life, reducing depressive symptoms, and suicidal ideation than psychotherapy or antidepressant treatment alone (Athanasiou et al., 2023; Kanani et al., 2025; Chen et al., 2021; Mazumder et al., 2023). Compared with conventional pharmacological treatments, these interventions demonstrate feasibility, safety, and cost-effectiveness profiles, reinforcing their potential for implementation in resource-constrained public health systems. Their delivery by trained multidisciplinary teams further supports their scalability and sustainability at the community level (Santoyo-Sánchez and Reyes-Morales, 2024; Apóstolo et al., 2016; Chen et al., 2021; Borbón-Castro et al., 2020; Cantón-Martínez et al., 2024; Ortiz-Ortiz et al., 2019).

In addition to exercise-based strategies, positive reminiscence interventions have consistently been shown to enhance life satisfaction, mood, social engagement, and cognitive outcomes (Apóstolo et al., 2016). Evidence from Mexico indicates reductions in depressive symptoms and improvements in the recall of specific positive memories following reminiscence-based programs, whereas other approaches, such as cognitive stimulation therapies, primarily contribute to maintaining or improving cognitive function (Villasán-Rueda et al., 2023; Sánchez-Nieto et al., 2019).

In contrast, evidence supporting video game–based and mental stimulation interventions remains limited and inconclusive (Gramaglia et al., 2021), with pilot studies in Mexico failing to demonstrate clear intervention-specific reductions in depressive symptomatology (Gómez-Miranda et al., 2019; Sánchez-Nieto et al., 2019). These heterogeneous findings highlight the need to critically differentiate between cognitively engaging activities and interventions capable of producing clinically meaningful emotional outcomes.

Globally, mental disorders affect approximately 15% of the geriatric population (Instituto Nacional de las Personas Adultas Mayores, 2024), yet access to adequate mental healthcare remains limited even in high-income countries. International evidence reveals a marked scarcity and uneven distribution of NPIs, with relatively few interventions identified over extended periods across Europe, North America, and low- and middle-income countries (Apóstolo et al., 2016; Xu et al., 2023; Wuthrich et al., 2024; Niclasen et al., 2018; Mazumder et al., 2023).

While several nations have implemented diverse, integrated strategies including mind–body practices, psychosocial stimulation, intergenerational learning, and nature-based or animal-assisted interventions (Gramaglia et al., 2021; Apóstolo et al., 2016; Xu et al., 2023; Reangsing et al., 2021; Frost et al., 2019; Niclasen et al., 2018; Mazumder et al., 2023; Wuthrich et al., 2024), Mexican programs remain largely concentrated on face-to-face modalities focused on physical exercise, cognitive stimulation, and reminiscence (Villarreal-Ángeles et al., 2016; Borbón-Castro et al., 2020; Cantón-Martínez et al., 2024; Gómez-Miranda et al., 2019; Ortiz-Ortiz et al., 2019; Sánchez-Nieto et al., 2019; Villasán-Rueda et al., 2023).

This limited diversity reflects structural constraints, including limited infrastructure, shortages of specialized human resources, and the absence of formal public policies that integrate NPIs into geriatric care models (Juárez et al., 2020; Guzmán-Olea et al., 2018).

Nevertheless, NPIs developed in Mexico demonstrate methodological rigor comparable to international studies, employing experimental or quasi-experimental designs and validated instruments to assess depression and life satisfaction (Gramaglia et al., 2021; Apóstolo et al., 2016; Xu et al., 2023; Niclasen et al., 2018; Mazumder et al., 2023; Wuthrich et al., 2024; Reangsing et al., 2021; Frost et al., 2019).

Most interventions in Mexico and around the world target non-institutionalized OAs, are of relatively short to moderate duration, and involve small sample sizes, highlighting persistent challenges related to scalability, long-term follow-up, and inclusion of more vulnerable subpopulations (Gramaglia et al., 2021; Reangsing et al., 2021; Frost et al., 2019; Niclasen et al., 2018; Mazumder et al., 2023; Apóstolo et al., 2016; Xu et al., 2023; Wuthrich et al., 2024; Ortiz-Ortiz et al., 2019; Villasán-Rueda et al., 2023).

In light of this evidence, the findings highlight the need to move beyond descriptive analyses toward policy-relevant, mechanism-informed frameworks. The integration of exercise-based and psychosocial NPIs into national aging and mental health strategies represents an opportunity to reduce treatment gaps and enhance emotional wellbeing and quality of life among OAs in Mexico.

## Challenges

Despite ample evidence supporting the benefits of NPIs on OA mental health (Gramaglia et al., 2021; Apóstolo et al., 2016; Xu et al., 2023; Reangsing et al., 2021; Frost et al., 2019; Villarreal-Ángeles et al., 2016; Borbón-Castro et al., 2020; Cantón-Martínez et al., 2024; Gómez-Miranda et al., 2019; Ortiz-Ortiz et al., 2019; Sánchez-Nieto et al., 2019; Villasán-Rueda et al., 2023; Niclasen et al., 2018; Mazumder et al., 2023; Wuthrich et al., 2024), significant challenges remain. The primary issue is the limited territorial coverage of NPIs, which are currently present in only 4 of Mexico’s 32 states (12.5%). Epidemiological evidence from Mexico indicates that only 12.7% of OA with depressive symptoms are detected, and merely 8.5% receive treatment. Moreover, increasing levels of municipal marginalization are associated with a widening gap between the prevalence of depressive symptoms and access to diagnosis and treatment. Consistent with the findings of the present review, these disparities are more pronounced in southern regions of the country than in Mexico City, suggesting insufficient institutional commitment to healthcare in rural and marginalized areas (Cerecero-García et al., 2020).

## Opportunities

The present review identifies clear opportunities to strengthen the implementation of NPIs in Mexico, supported by national clinical guidelines that endorse a biopsychosocial approach to depression in OAs (Instituto Mexicano del Seguro Social & Centro Nacional de Excelencia Tecnológica en Salud, 2010). Community, academic, and institutional settings may serve as scalable, cost-effective platforms for integrating culturally adapted, evidence-based NPIs. However, current implementation remains constrained by weak interdisciplinary coordination (Consejo Mexicano de Geriatría, 2025; Santoyo-Sánchez and Reyes-Morales, 2024). To maximize impact, future efforts should prioritize inclusive models that address OAs with functional dependence or chronic conditions, ensuring continuity of care across home-based and institutional settings. Such an approach underscores the need for immediate action to prioritize equitable, inclusive, and wellness-centered models of geriatric care.

## Limitations

The findings of this review should be interpreted in light of several limitations related to both the primary studies and the review process itself. Regarding the included evidence, the methodological quality was variable. Two studies were randomized controlled trials (Villarreal-Ángeles et al., 2016; Borbón-Castro et al., 2020), and five were quasi-experimental (Cantón-Martínez et al., 2024; Gómez-Miranda et al., 2019; Ortiz-Ortiz et al., 2019; Sánchez-Nieto et al., 2019; Villasán-Rueda et al., 2023). The mean quality scores were 5.5 (maximum 13) and 6.8 (maximum 9), respectively.

The most frequent contributors to methodological limitations were a lack of blinding, small sample sizes, short intervention duration, and limited control of confounding variables. While blinding is inherently difficult in physical interventions (Adams et al., 2021), these scores indicate a moderate risk of bias, warranting cautious interpretation of efficacy. Consequently, the observed effects should be interpreted as suggestive rather than definitive. Overall confidence in the magnitude of the reported benefits is moderate. Furthermore, most interventions target non-institutionalized OAs, are of short duration, and involve small sample sizes, highlighting persistent challenges related to long-term follow-up and inclusion of vulnerable subpopulations (Gramaglia et al., 2021; Reangsing et al., 2021; Frost et al., 2019; Niclasen et al., 2018; Mazumder et al., 2023; Apóstolo et al., 2016; Xu et al., 2023; Wuthrich et al., 2024; Ortiz-Ortiz et al., 2019; Villasán-Rueda et al., 2023).

Regarding the review process, two specific limitations must be acknowledged. First, the search strategy was restricted to PubMed, Scopus, and the Virtual Health Library. While these databases provide comprehensive coverage of high-impact medical literature, their exclusion of other databases (e.g., Embase, PsycINFO) and grey literature may have led to the omission of relevant local reports or unpublished dissertations. Second, the search algorithm used English and Spanish terms but may have introduced linguistic bias by excluding studies published in indigenous languages or in journals not indexed in major repositories (Dobrescu et al., 2021). Moreover, Dobrescu et al. note that restricting systematic reviews to English-language publications minimally affects estimates and conclusions for most medical topics (Dobrescu et al., 2021).

## Conclusion

Taken together, the findings of this review suggest that NPIs constitute a promising yet underutilized strategy to promote, maintain, and improve mental health among OAs in Mexico. Their limited and uneven implementation reflects persistent structural gaps within the health system and broader societal neglect of geriatric care. Addressing these challenges requires the development of robust, inclusive, and sustainable public policies that support the integration of culturally relevant, non-pharmacological psychosocial interventions into routine care. Future research must move beyond pilot studies to prioritize larger, nationally representative samples and explicitly include OAs with multimorbidity, functional dependence, or sensory impairments. Only through such rigorous and scalable approaches can geriatric mental healthcare evolve from a privilege into a standardized, essential component of the national health system.

## Data Availability

The original contributions presented in the study are included in the article/Supplementary Material, further inquiries can be directed to the corresponding author.
