# Supplementary material for: Non-pharmacological mental health interventions for older adults in Mexico: a systematic review
Source: Front Aging. 2026 Feb 23;7:1730672. doi: 10.3389/fragi.2026.1730672 (PMC12980019; doi:10.3389/fragi.2026.1730672)
Supplement: Supplementary file 1 [file Table1.docx]

**Supplementary 1**

*Excluded studies by reason*

| **Wrong outcome** |
| --- |
| 1. Mendoza-Ruvalcaba, N. M., & Fernández-Ballesteros, R. (2016). Effectiveness of the vital aging program to promote active aging in Mexican older adults. *Clinical interventions in aging*, *11*, 1631–1644. <https://doi.org/10.2147/CIA.S102930>  2. Villasán-Rueda, A., Sánchez-Cabaco, A., Mejía-Ramírez, M., Afonso, R. M., & Castillo-Riedel, E. (2023). Transcultural Pilot Study of the Efficacy of Reminiscence Therapy for Mexican and Spanish Older Adults with Different Levels of Cognitive Decline. *Journal of cross-cultural gerontology*, *38*(4), 371–388. <https://doi.org/10.1007/s10823-023-09486-2> |
| **Wrong population** |
| 3. Aoki Morantte, A. S., Medina-Rivera, M. V., & Nicolini, H. (2024). Programa de estimulación cognitiva en línea para pacientes adultos mayores con deterioro cognitivo leve: estudio de factibilidad. *Revista espanola de geriatria y gerontologia*, *59*(6), 101526. https://doi.org/10.1016/j.regg.2024.101526  4. Perkins, K. M., Munguia, N., Angulo, A., Anaya, C., Rios, R., & Velazquez, L. (2021). Evaluation of aquafitness exercise on the physical and mental health of older women: a pilot study. *Journal of women & aging*, *33*(6), 569–582. <https://doi.org/10.1080/08952841.2020.1730681>  5. Sánchez Mascuñano, A., Lapena Estella, C., Continente García, X., Laguna Fernández, V., del Val García, J. L., & López Medina, M. J. (2019). Evaluación de una intervención de promoción de la salud para personas mayores de barrios desfavorecidos. *Metas de Enfermería, 22*(7), 24-32. <https://doi.org/10.35667/MetasEnf.2019.22.1003081464> |
| **Wrong design** |
| 6. Merizzi, A., Biasi, R., Zamudio, J. F. Á., Spagnuolo Lobb, M., Di Rosa, M., & Santini, S. (2022). A Single-Case Design Investigation for Measuring the Efficacy of Gestalt Therapy to Treat Depression in Older Adults with Dementia in Italy and in Mexico: A Research Protocol. *International journal of environmental research and public health*, *19*(6), 3260. <https://doi.org/10.3390/ijerph19063260>  7. Ronquillo, L., Zamudio, V., Gutiérrez-Hernández, D., Lino, C., Navarro, J., & Doctor, F. (2020). *Towards an automatic recommendation system to well-being for elderly based on augmented reality*. In *2020 16th International Conference on Intelligent Environments (IE)* (pp. 126–131). IEEE. https://doi.org/10.1109/IE49459.2020.9155010 |
